# Designing thermophilic, synthetic microbial communities for consolidated bioprocessing

**DOI:** 10.1016/j.bidere.2025.100024

**Published:** 2025-04-21

**Authors:** Hansen Tjo, Kelly Blundin, Jonathan M. Conway

**Affiliations:** a Department of Chemical and Biological Engineering, Princeton University, Princeton, NJ, 08544, USA; b Department of Molecular Biology, Princeton University, Princeton, NJ, 08540, USA; c Omenn-Darling Bioengineering Institute, Princeton University, Princeton, NJ, 08544, USA; d Andlinger Center for Energy and the Environment, Princeton University, Princeton, NJ, 08544, USA; e High Meadows Environmental Institute, Princeton University, Princeton, NJ, 08544, USA

**Keywords:** Thermophile, Co-cultures, Microbial communities, Consolidated bioprocessing, Lignocellulose, Substrate specialization, Division of labor, Biofuel

## Abstract

Lignocellulose-derived fuels and chemicals are vital to breaking the world's dependence on fossil fuels. Though plant biomass is notoriously resistant to deconstruction, lignocellulolytic thermophiles are especially adept at degrading its constituent polysaccharides into mono- and oligo-saccharides for catabolism. And many thermophiles, whether lignocellulolytic or not, can be engineered to ferment lignocellulose-derived sugars into valuable fuels and chemicals as part of consolidated bioprocesses. Although the past twenty years have seen major advances in the genetic and metabolic engineering of individual thermophiles, the strategy of co-culturing thermophilic strains as part of synthetic communities has not been well established. Synthetic communities unlock synergistic interactions that outperform monocultures, thereby enhancing product titers, rates, and yields. While limited genetic tools once hindered the development of synthetic thermophilic communities, recent advances now offer robust systems for engineering these industrially relevant organisms. Here, we propose that this expanded genetic malleability enables engineering of 1) transport specialization to reduce substrate competition between strains and 2) division of labor strategies whereby one strain focuses on lignocellulose deconstruction while another strain dedicates metabolic burden for product synthesis. We draw on examples of engineered thermophiles like *Clostridium thermocellum, Thermoanaerobacter saccharolyticum,* and *Anaerocellum bescii* to illustrate how these mechanisms have been applied in thermophilic co-cultures. In brief, this perspective outlines design principles to construct effective thermophilic communities for lignocellulose bioprocessing.

## 1 Introduction

Biofuels and biochemicals from renewable feedstocks offer sustainable alternatives to their fossil fuel derived counterparts [1,2]. Thermophilic, lignocellulosic biomass-degrading microbes can play a crucial role in displacing petroleum-based supply chains given their ability to overcome biomass recalcitrance and produce valuable products such as ethanol from renewable feedstocks [[3], [4], [5]]. These thermophiles operate at elevated temperatures (55–100 ​°C), yielding several process advantages including enhanced reaction kinetics, reduced risks of bioreactor contamination, and lower energy costs for temperature control [4,[6], [7], [8], [9]]. Engineered strains can also perform consolidated bioprocessing whereby biomass degradation and sugar fermentation are combined in a single step, eliminating the need for separate saccharification and fermentation process stages [10,11].

Meanwhile, microbial co-cultures have been deployed as a biotechnology strategy to increase chemical titers, rates, and yields by expanding metabolic capability [[12], [13], [14], [15]]. Historically, efforts to integrate lignocellulolytic thermophiles as part of co-culture processes have been stymied by underdeveloped genetic engineering toolkits in these non-model microbes [11,12,[16], [17], [18], [19]]. Their requirement of high temperature growth conditions meant limited access to functional genetic parts and tools (e.g., antibiotic resistance markers, fluorescent reporters, transposons, CRISPR-Cas9 systems) typically only active under mesophilic conditions. Yet, recent advancements have produced a surge in new genetic parts, easing genetic modifications for many lignocellulolytic and other industrially relevant thermophiles [[20], [21], [22], [23], [24]]. These advancements in turn make co-culturing a broader array of engineered thermophilic strains a newly viable synthetic biology strategy, for which we discuss design principles to maximize their efficacy in bioprocessing contexts.

## 2 Thermophilic Co-culture Strategies

### 2.1 Engineering thermophilic synthetic communities via substrate specialization

**Fig. 1:**
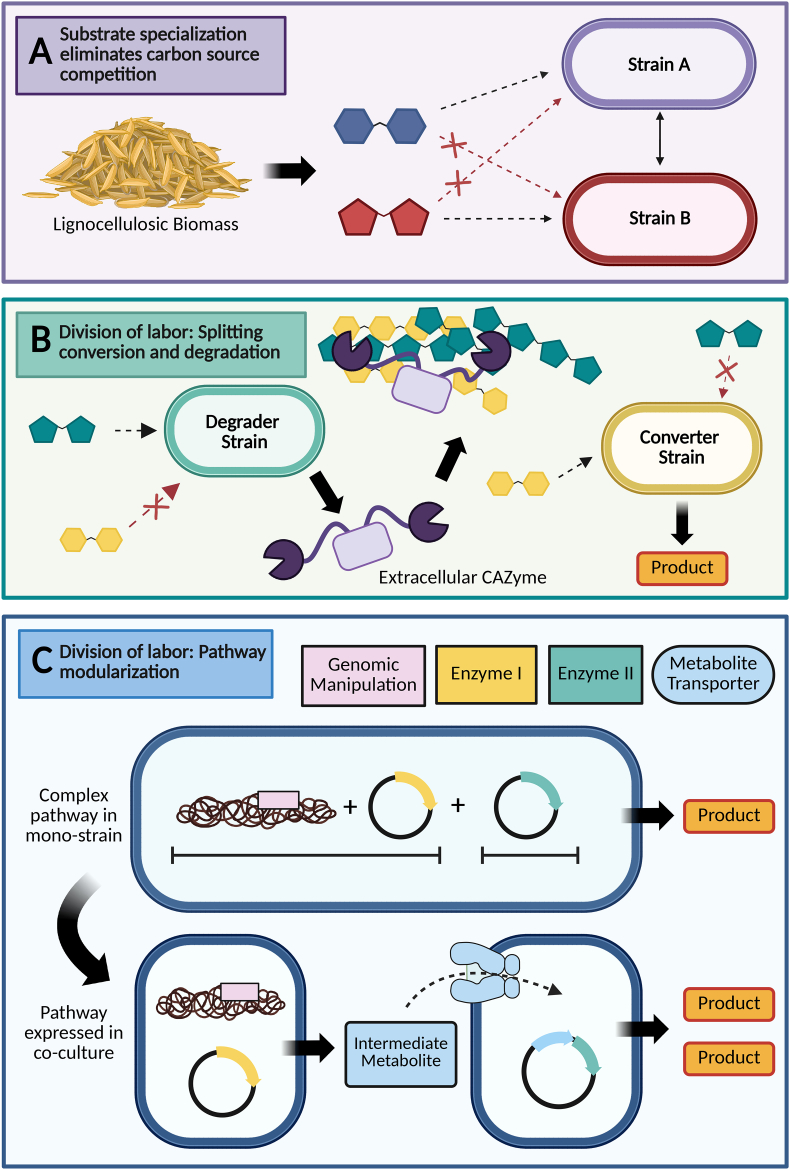
**Biodesign strategies for synthetic, thermophilic communities.** A) Carbon substrate specialization is one strategy basis for engineered thermophilic communities. Microbes can be genetically engineered for removal of specialized substrate uptake pathways, eliminating competition with other thermophiles in co-culture. Here, to avoid substrate competition, thermophilic microbes Strain A and Strain B grow on distinct sugars from lignocellulosic biomass. B) Synthetic thermophilic communities can leverage division of labor for more efficient consolidated bioprocessing of lignocellulosic biomass. “Degrader” strains are specialized for deconstruction of lignocellulosic biomass, while “Converter” strains, which are minimally involved in biomass degradation, instead focus on production of value-added chemicals. Splitting the roles of biomass degradation and conversion decreases the metabolic burden placed on each strain, improving bioprocessing efficiency. C) Complex biosynthetic pathways for bioproduction can be modularized across multiple strains. In the given example, a single strain is overburdened by multiple genetic manipulations i.e. one perturbation to its genome and expression of two heterologous enzymes (Enzyme I and Enzyme II) to generate the desired bioproduct. Burden sharing these genetic modifications across two strains, while ensuring any pathway intermediate can be transported into the strain containing the final production step, can result in higher product titers (Created in Biorender: https://BioRender.com/ggd42hw).

Lignocellulose deconstruction produces a diverse range of carbohydrates, including pentose and hexose sugars in various oligomeric forms. To maximize biomass conversion, co-culture strains should collectively consume this expansive range of substrates. In nature, lignocellulolytic thermophiles such as *Clostridium thermocellum*, *Anaerocellum bescii,* and various *Thermoanaerobacter* species scavenge in hot springs and other thermal environments for carbon sources. Many consequently possess diverse sugar transporters and utilization pathways. Therefore, co-cultures containing substrate-specialized strains can be tailored around native sugar preferences, or by engineering strains through modifications of sugar transporters (adding exogenous ones or deleting native ones) and metabolic pathways [25,26]. Such tailored substrate utilization not only maximizes product yields but also improves community stability by eliminating growth source competition (Fig. 1A).

The *C. thermocellum* and *Thermoanaerobacter saccharolyticum* co-culture is a model example of leveraging native substrate specialization, as *C. thermocellum* effectively uptakes cello-oligosaccharides (and cannot utilize xylose or arabinose), while *T. saccharolyticum* can ferment pentose sugars released from hemicellulose [[27], [28], [29]]. Consumption of pentose sugars by *T. saccharolyticum* also prevents end-product inhibition of *C. thermocellum* hemicellulases; in other words, improved biomass hydrolysis is dependent on the growth of both thermophiles [30]. Another example includes *Thermoanaerobacterium aotearoense* SCUT27 that has been modified to uptake and metabolize sucrose [31]. As *C. thermocellum* cannot consume sucrose or pentose sugars, but can consume hexose sugars, co-culturing it with *T. aotearoense* SCUT27 fully utilizes sugars from both lignocellulosic and sucrose-rich agricultural feedstocks [31,32]. Engineered strains of *A. bescii* that cannot utilize starch or cellulose, relying solely on hemicellulose for growth, could likewise be co-cultured with *C. thermocellum* [33,34].

### 2.2 Synthetic thermophilic communities can leverage a “division of labor” to maximize metabolic efficiency

Synthetic thermophilic communities are well-suited to deconstructing the complex polysaccharide diversity of plant biomass. In these communities, individual thermophilic strains can enjoy reduced metabolic burden by specializing in synergistic, strain-specific functions that increase growth rates, product yields, and overall metabolic efficiency of the co-culture [25,[35], [36], [37]]. For example, different thermophilic strains possess distinct glycoside hydrolase (GH) inventories with functional complementarity in degrading cellulose and the diverse polysaccharides that make up hemicellulose (e.g., arabinans, xylans) [7,25]. And, even in organisms with enzyme inventories containing similar GH families, differences in enzyme expression, kinetics, and synergism affect the efficiency of lignocellulose degradation [25]. The engineered co-culture of *C*. *thermocellum* and engineered *T*. *saccharolyticum* remains a salient representation of synergy between enzyme inventories across a thermophilic community: while *C. thermocellum* can solubilize over 90 ​% of glucuronoarabinoxylan (GAX) from corn fiber, *T. saccharolyticum* cannot effectively ferment the released oligosaccharides [20]. Successful fermentation of these GAX oligosaccharides depended on the addition of glycoside hydrolases from *Herbinix* spp. strain LL1355 into engineered *T. saccharolyticum,* enabling this co-culture system — leveraging enzymes from three different thermophilic species — to achieve greater levels of GAX utilization and ethanol production [27].

Another common division of labor approach is to simply engineer complementarity by splitting roles involving biomass deconstruction and bioproduct conversion across different strains (Fig. 1B). Strains could be engineered to specialize in extracellular CAZymes expression without generating desired chemical products, or the production of a desired chemical coupled with reduced CAZyme expression. This approach could be applied to the previously mentioned co-culture involving *C. thermocellum* and *T. thermosaccharolyticum* [27]. Though *T. thermosaccharolyticum* is the dedicated ethanol-generator in this co-culture, it experiences unnecessary metabolic burden from producing its native CAZymes when *C. thermocellum* is already responsible for plant biomass deconstruction. A modification of this community could entail *C. thermocellum* expressing all necessary CAZymes for substrate solubilization, freeing *T. thermosaccharolyticum* to solely focus on ethanol production. And although CAZyme genes are typically scattered throughout thermophile genomes, implying multiple deletions are necessary to meaningfully reduce metabolic burden, targeting select highly transcribed CAZyme loci for deletion may suffice [[38], [39], [40], [41], [42]]. *Anaerocellum bescii*'s Glucan-Degradation-Locus (GDL) is largely responsible for its lignocellulolytic activity, and its deletion has been shown to substantially lower its secretome's protein content [42,43]. And, the role of biomass degradation can also be further divided into distinct strains. Co-culturing *A. bescii* strains, each expressing a subset of CAZyme from its GDL, achieved the same biomass degradation as a wild-type monoculture with a complete GDL, while reducing individual metabolic burden [42,43].

Although more challenging to implement, synthetic thermophilic communities can also be harnessed to modularize complex biosynthetic pathways (Fig. 1C). By distributing metabolic pathways more evenly across constituent strains – as opposed to expression of all pathway components within a single strain – this division of labor offers a synergistic advantage by enhancing the fitness of each strain [16,17,25]. Moreover, this modularization process also enables separate optimization of each individual reaction, and avoids overburdening a single strain with numerous iterative genetic modifications that may compromise genome stability. To our knowledge this effort has only been successfully demonstrated in mesophiles; for example, Zhang et al. (2015) demonstrated this in a co-culture of distinct *Escherichia coli* strains for the production of 4-hydroxybenzoic acid [12]. Despite the challenges of implementing metabolic handoffs between co-cultured strains to separate metabolic pathways, this approach may become more viable for thermophiles in the future.

It is worth noting that thermophilic archaea – notably *Sulfolobus solfataricus* and *Pyrococcus furiosus* – face limitations to lignocellulose bioprocessing co-cultures. *S. solfataricus* is attractive for substrate transport modification due to its broad sugar uptake capabilities and availability of genetic tools [[44], [45], [46]]. It possesses multiple ABC sugar transporters for the uptake of disaccharides such as cellobiose and maltose, and lignocellulose-derived monosaccharides such as glucose and arabinose [[44], [45], [46], [47]]. Despite this, successful co-cultures leveraging *S. solfataricus* must accommodate its acidophilic nature, lengthy doubling times, and absence of fuel- and chemical-producing metabolic pathways for division of labor strategies [45]. While *P. furiosus* has been successfully engineered to produce multiple commodities such as ethanol, butanol, acetoin, and 3-hydroxypropionic acid (3-HP), its substrate utilization range is limited [[48], [49], [50], [51], [52], [53]]. *P. furiosus* primarily grows on starch-derived oligosaccharides and cannot grow on most lignocellulosic sugars [[48], [49], [50],^53^]. Presently, their thermophilic bacterial counterparts remain more suitable candidates for bioprocessing co-cultures for the conversion of lignocellulosic substrates.

While this perspective primarily focuses on lignocellulolytic thermophiles, not every member of a synthetic thermophilic community needs to degrade plant biomass. For example, thermophilic *Geobacillus* strains, such as *Geobacillus thermoglucosidasius*, which have been engineered to produce ethanol and lactic acid, can rely on dedicated lignocellulose-degrading strains to provide the hexose and pentose monosaccharides it needs for growth [54,55]. Ultimately, we envision that the choice of organisms in industrially deployable thermophilic synthetic communities will be driven by the ability to tailor their abilities (biomass degradation, sugar transport, metabolism, etc.) through improved genetic manipulation.

## 3 Conclusion

In sum, we outline two design strategies for thermophilic co-cultures: substrate specialization to enhance consortia stability and maximize biomass utilization (Fig. 1A) and division of labor to reduce metabolic burden (Fig. 1B and C). As these strategies are not mutually exclusive, they could be combined to achieve maximum benefit. It is no surprise many of our co-culture examples involve *C. thermocellum, A. bescii,* and *Thermoanaerobacter* species given their broad strengths across complementary CAZyme profiles, compatible growth temperatures, and broad substrate flexibility. We are optimistic that combining these co-culture strategies with new genetic advances in thermophilic microbes will further the production of renewable fuels and chemicals from lignocellulose at industrial scale.

## Author contributions

H.T. and J.M.C. conceived of the work, H.T., K.B., J.M.C wrote and edited the article, H.T. and K.B. prepared the figure.

## Funding

This work has been supported by the 10.13039/100020601High Meadows Environmental Institute at Princeton University through the generous support of the William Clay Ford, Jr ‘79 and Lisa Vanderzee Ford ‘82 Graduate Fellowship Fund to H.T.; by a Roberto Rocca Graduate Fellowship from Techint Group to H.T., and a grant from the Energy Research Fund administered by the 10.13039/100019333Andlinger Center for Energy and the Environment at Princeton University to J.M.C.

## Declaration of competing interest

The authors declare that they have no known competing financial interests or personal relationships that could have appeared to influence the work reported in this paper.

## References

[1] Fulton L.M., Lynd L.R., Körner A., Greene N., Tonachel L.R. (2015). The need for biofuels as part of a low carbon energy future. Biofuels, Bioproducts and Biorefining.

[2] Hannula I., Reiner D.M. (Oct. 2019). Near-term potential of biofuels, electrofuels, and battery electric vehicles in decarbonizing road transport. Joule.

[3] Loder A.J., Biotechnology Industrial, Wittmann C., Liao J.C. (2016). Extreme Thermophiles as Metabolic Engineering Platforms: Strategies and Current Perspective.

[4] Crosby J.R., Laemthong T., Lewis A.M., Straub C.T., Adams M.W., Kelly R.M. (Oct. 2019). Extreme thermophiles as emerging metabolic engineering platforms. Curr. Opin. Biotechnol..

[5] Gilna P., Lynd L.R., Mohnen D., Davis M.F., Davison B.H. (Dec. 2017). Progress in understanding and overcoming biomass recalcitrance: a BioEnergy Science Center (BESC) perspective. Biotechnol. Biofuels.

[6] Jiang Y., Jiang W., Xin F., Zhang W., Jiang M. (Jun. 2022). Thermophiles: potential chassis for lignocellulosic biorefinery. Trends Biotechnol..

[7] Blumer-Schuette S.E. (2014). Thermophilic lignocellulose deconstruction. FEMS Microbiol. Rev..

[8] Bing R.G. (Jan. 2023). Fermentative conversion of unpretreated plant biomass: a thermophilic threshold for indigenous microbial growth. Bioresour. Technol..

[9] Adams M.W.W., Kelly R.M. (Dec. 2016). The renaissance of life near the boiling point – at last, genetics and metabolic engineering. Microb. Biotechnol..

[10] Singhania R.R. (Jun. 2022). Consolidated bioprocessing of lignocellulosic biomass: technological advances and challenges. Bioresour. Technol..

[11] Argyros D.A. (Dec. 2011). High ethanol titers from cellulose by using metabolically engineered thermophilic, anaerobic microbes. Appl. Environ. Microbiol..

[12] Zhang H., Pereira B., Li Z., Stephanopoulos G. (Jul. 2015). Engineering Escherichia coli coculture systems for the production of biochemical products. Proc. Natl. Acad. Sci..

[13] Cui Y., Yang K.-L., Zhou K. (Sep. 2021). Using Co-culture to functionalize Clostridium fermentation. Trends Biotechnol..

[14] Tsoi R., Wu F., Zhang C., Bewick S., Karig D., You L. (Mar. 2018). Metabolic division of labor in microbial systems. Proc. Natl. Acad. Sci..

[15] Lindemann S.R. (Sep. 2016). Engineering microbial consortia for controllable outputs. ISME J..

[16] Bailey J.E. (1991). Toward a science of metabolic engineering. Science.

[17] Nielsen J., Keasling J.D. (Mar. 2016). Engineering cellular metabolism. Cell.

[18] Duncker K.E., Holmes Z.A., You L. (Nov. 2021). Engineered microbial consortia: strategies and applications. Microb. Cell Fact..

[19] Jiang Y., Wu R., Zhang W., Xin F., Jiang M. (Nov. 2023). Construction of stable microbial consortia for effective biochemical synthesis. Trends Biotechnol..

[20] Ganguly J., Martin-Pascual M., van Kranenburg R. (Dec. 2019). CRISPR interference (CRISPRi) as transcriptional repression tool for Hungateiclostridium thermocellum DSM 1313. Microb. Biotechnol..

[21] Le Y., Fu Y., Sun J. (Dec. 2020). Genome editing of the anaerobic thermophile thermoanaerobacter ethanolicus using thermostable Cas9. Appl. Environ. Microbiol..

[22] Adalsteinsson B.T. (May 2021). Efficient genome editing of an extreme thermophile, Thermus thermophilus, using a thermostable Cas9 variant. Sci. Rep..

[23] Lipscomb G.L., Conway J.M., Blumer-Schuette S.E., Kelly R.M., Adams M.W.W. (Jun. 2016). A highly thermostable kanamycin resistance marker expands the tool kit for genetic manipulation of caldicellulosiruptor bescii. Appl. Environ. Microbiol..

[24] Groom J., Chung D., Young J., Westpheling J. (Sep. 2014). Heterologous complementation of a pyrF deletion in Caldicellulosiruptor hydrothermalis generates a new host for the analysis of biomass deconstruction. Biotechnol. Biofuels.

[25] Lindemann S.R. (Dec. 2020). A piece of the pie: engineering microbiomes by exploiting division of labor in complex polysaccharide consumption. Current Opinion in Chem. Eng..

[26] Tjo H., Conway J.M. (Jun. 2024). Sugar transport in thermophiles: bridging lignocellulose deconstruction and bioconversion. J. Ind. Microbiol. Biotechnol..

[27] Beri D., York W.S., Lynd L.R., Peña M.J., Herring C.D. (Apr. 2020). Development of a thermophilic coculture for corn fiber conversion to ethanol. Nat. Commun..

[28] Zambello I.U., Holwerda E.K., Lynd L.R. (Aug. 2024). Characterization of sugarcane bagasse solubilization and utilization by thermophilic cellulolytic and saccharolytic bacteria at increasing solid loadings. Bioresour. Technol..

[29] Pang J., Hao M., Li Y., Liu J., Lan H., Zhang Y., Zhang Q., Liu Z. (Sep. 2018). Consolidated bioprocessing using *Clostridium thermocellum* and *Thermoanaerobacterium thermosaccharolyticum* co-culture for enhancing ethanol production from corn straw. BioRes.

[30] Liu Y., Yu P., Song X., Qu Y. (Jun. 2008). Hydrogen production from cellulose by co-culture of Clostridium thermocellum JN4 and Thermoanaerobacterium thermosaccharolyticum GD17. Int. J. Hydrogen Energy.

[31] Dai K., Qu C., Feng J., Lan Y., Fu H., Wang J. (Oct. 2023). Metabolic engineering of Thermoanaerobacterium aotearoense strain SCUT27 for biofuels production from sucrose and molasses. Biotechnol. Biofuel. Bioprod..

[32] Yan F. (2022). Deciphering cellodextrin and glucose uptake in Clostridium thermocellum. mBio.

[33] Tjo H., Jiang V., Calvo A., Joseph J.A., Conway J.M. (2025). “A highly conserved ABC transporter mediates cello-oligosaccharide uptake in the extremely thermophilic. Lignocellulolytic Bacterium Anaerocellum (f. Caldicellulosiruptor) bescii,”.

[34] Tjo H., Jiang V., Joseph J.A., Conway J.M. (2024). “Maltodextrin transport in the extremely thermophilic. Lignocellulose-Degrading Bacterium Anaerocellum bescii (f. Caldicellulosiruptor bescii),” bioRxiv.

[35] Jia X. (Jun. 2016). Design, analysis and application of synthetic microbial consortia. Synthetic and Sys. Biotechnol..

[36] Minty J.J. (Sep. 2013). Design and characterization of synthetic fungal-bacterial consortia for direct production of isobutanol from cellulosic biomass. Proc. Natl. Acad. Sci..

[37] Brenner K., You L., Arnold F.H. (Sep. 2008). Engineering microbial consortia: a new frontier in synthetic biology. Trends Biotechnol..

[38] Rodionov D. (2013). Transcriptional regulation of the carbohydrate utilization network in Thermotoga maritima. Front. Microbiol..

[39] Voorhorst W.G.B. (Jun. 1999). Transcriptional regulation in the hyperthermophilic archaeon Pyrococcus furiosus: coordinated expression of divergently oriented genes in response to β-linked glucose polymers. J. Bacteriol..

[40] Vanfossen A.L., Verhaart M.R.A., Kengen S.M.W., Kelly R.M. (Dec. 2009). Carbohydrate utilization patterns for the extremely thermophilic bacterium Caldicellulosiruptor saccharolyticus reveal broad growth substrate preferences. Appl. Environ. Microbiol..

[41] Rodionov D.A. (Jun. 2021). Transcriptional regulation of plant biomass degradation and carbohydrate utilization genes in the extreme thermophile *Caldicellulosiruptor bescii*. mSystems.

[42] Conway J.M. (Dec. 2017). Functional analysis of the glucan degradation locus in caldicellulosiruptor bescii reveals essential roles of component glycoside hydrolases in plant biomass deconstruction. Appl. Environ. Microbiol..

[43] Conway J.M., Crosby J.R., McKinley B.S., Seals N.L., Adams M.W.W., Kelly R.M. (2018). Parsing in vivo and in vitro contributions to microcrystalline cellulose hydrolysis by multidomain glycoside hydrolases in the Caldicellulosiruptor bescii secretome. Biotechnol. Bioeng..

[44] Elferink M.G.L., Albers S.-V., Konings W.N., Driessen A.J.M. (2001). Sugar transport in Sulfolobus solfataricus is mediated by two families of binding protein-dependent ABC transporters. Mol. Microbiol..

[45] Quehenberger J., Shen L., Albers S.-V., Siebers B., Spadiut O. (Dec. 2017). Sulfolobus – a potential key organism in future biotechnology. Front. Microbiol..

[46] Ulas T., Riemer S.A., Zaparty M., Siebers B., Schomburg D. (Aug. 2012). Genome-scale reconstruction and analysis of the metabolic network in the hyperthermophilic archaeon Sulfolobus solfataricus. PLoS One.

[47] Wolf J. (Dec. 2016). A systems biology approach reveals major metabolic changes in the thermoacidophilic archaeon Sulfolobus solfataricus in response to the carbon source L-fucose versus D-glucose. Mol. Microbiol..

[48] Vailionis J.L. (Jun. 2023). Optimizing strategies for bio-based ethanol production using genome-scale metabolic modeling of the hyperthermophilic archaeon, Pyrococcus furiosus. Appl. Environ. Microbiol..

[49] Lipscomb G.L. (Jun. 2023). Manipulating fermentation pathways in the hyperthermophilic archaeon Pyrococcus furiosus for ethanol production up to 95°C driven by carbon monoxide oxidation. Appl. Environ. Microbiol..

[50] Nguyen D.M.N. (Mar. 2016). Temperature-dependent acetoin production by Pyrococcus furiosus is catalyzed by a biosynthetic acetolactate synthase and its deletion improves ethanol production. Metab. Eng..

[51] Keller M.W., Lipscomb G.L., Loder A.J., Schut G.J., Kelly R.M., Adams M.W.W. (Jan. 2015). A hybrid synthetic pathway for butanol production by a hyperthermophilic microbe. Metab. Eng..

[52] Keller M.W. (Apr. 2013). Exploiting microbial hyperthermophilicity to produce an industrial chemical, using hydrogen and carbon dioxide. Proc. Natl. Acad. Sci..

[53] Kengen S.W.M. (Feb. 2017). Pyrococcus furiosus, 30 years on. Microb. Biotechnol..

[54] Cripps R.E., Eley K., Leak D.J., Rudd B., Taylor M., Todd M. (Nov. 2009). Metabolic engineering of *Geobacillus thermoglucosidasius* for high yield ethanol production. Metab. Eng..

[55] Liu J., Han X., Tao F., Xu P. (Feb. 2024). Metabolic engineering of *Geobacillus thermoglucosidasius* for polymer-grade lactic acid production at high temperature. Bioresour. Technol..

